# Diversity on the bench: An analysis of gendered biases in the language of Australian Family Law Court judgments

**DOI:** 10.1371/journal.pone.0331841

**Published:** 2025-09-08

**Authors:** Elma Akand, Yanan Fan, Wayne Wobcke, Scott A. Sisson, Mehera San Roque

**Affiliations:** 1 School of Mathematics and Statistics and UNSW Data Science Hub, UNSW, Sydney, New South Wales, Australia; 2 Data61, CSIRO, Eveleigh, New South Wales, Australia; 3 School of Computer Science and Engineering, UNSW, Sydney, New South Wales, Australia; 4 School of Law, Society and Criminology, UNSW, Sydney, New South Wales, Australia; Southwestern University of Finance and Economic, CHINA

## Abstract

In this paper we analyse gender-based biases in the language within complex legal judgments. Our aims are: (i) to determine the extent to which purported biases discussed in the literature by feminist legal scholars are identifiable from the language of legal judgments themselves, and (ii) to uncover new forms of bias represented in the data that may promote further analysis and interpretation of the functioning of the legal system. We consider a large set of 2530 judgments in family law in Australia over a 20 year period, examining the way that male and female parties to a case are spoken to and about, by male and female judges, in relation to their capacity to provide care for children subject to the decision. Structural topic modelling is used to develop coherent topics for sentences that fall under the notion of “capacity”, which are further differentiated by the gender of both the target of the sentence and the gender of the judge. The analysis reveals significant gendered differences in the language used in these documents, determined by both the gender of the target and the gender of the judge.

## Introduction

The existence of gendered and discriminatory decision making in the law has been well established and extensively analysed [1,2]. Common Law countries of the global North, such as Australia, the United States, Canada and England have historically excluded women from accessing the same rights, protections and benefits as men, for example, by restricting access to certain professions, including the legal profession [3], or presumptions that it was not possible for a husband to rape his wife [4].

While *explicitly* discriminatory legal rules have generally been removed or reformed, the effects of gendered social expectations and structural inequalities remain embedded in the operation of the legal system [5]. Gendered differences and social biases are reflected in, and constituted by, legal practice, texts and language, just as they are reflected in language use and social interactions more broadly [6]. For example, a recent study found that gender essentialism (that is, the belief in an inherent, immutable gendered and racialized identity [7]) plays a role in shaping negative reactions towards individuals who defy traditional gender norms [8]. Significantly, this study noted that in the context of political power dynamics, backlash was not limited to penalizing power-seeking women but also extended to men who do not exhibit power-seeking behaviour, revealing the deep-seated nature of gender norm expectations as well as resistance to changing these norms. Similar patterns can be observed in legal cases where defendants are perceived to have failed to meet gendered social expectations around motherhood [9], or where witnesses/complainants may be perceived to lack credibility when there has been a delay in reporting a sexual assault [10]. Analysis has even identified differences in case outcome associated with a legal *advocate’s* compliance or non-compliance with gendered norms [11].

Since social inequalities can be reinforced by gendered or discriminatory expression and interactions, understanding how such discrimination or “bias”, is expressed in legal documents, proceedings and judgments is important for understanding how law and the legal system operates to reinforce gender (and other) inequalities [12,13]. A reliance on judge-made law is a distinctive feature of the legal system in Common Law countries [14]. A judge making a decision and explaining the reasons for that decision in their judgment will necessarily be drawing on their own life experiences, training and environment (whether they are conscious of this, or articulate this, or not) [15]. Consequently, there has been considerable attention paid to both the impact that a lack of gender (and other) diversity may have on the nature and development of legal rules themselves across multiple legal domains [2]. While judges (and other participants in the legal system) may no longer express their gender bias as explicitly as they may have in the past, more subtle expressions of gendered differences and bias persist. For example, previous work has shown that gender will impact the ways that the credibility of witnesses will be described and judged by decision makers [16,17], or in the ways that loss and damages will be described and assessed for female vs male claimants in personal injury cases [18,19]. The gendered nature of the legal profession may impact not just the development of law, and the diversity of the profession per se, but also perceptions of who can speak authoritatively as a judge [14,15] or as a lawyer. For example, a survey in the New York courts identified persistent and widespread gender bias in the reception of arguments put by female advocates [20], and recent work has also identified gendered differences in the ways that lawyers and even US Supreme Court and Australian High Court judges are interrupted during oral hearings [21,22], and see [23]. Up until recently, identifying or exploring gender or other biases in the language used in judgments and other legal texts relied primarily on content or discourse analysis, informed by a feminist (or related) critical framework [24–26]. More recently, as discussed further below, methods that allow for analysis of larger datasets supported by some automation have been introduced. Our study draws on the insights derived from traditional content, discourse and linguistic analysis but extends this to a much larger dataset than has traditionally been possible.

In parallel with this line of thought, the increased use of machine learning methods (including Large Language Models), which are trained using existing documents can embed and replicate biases inherent in language. This should be a cause for concern, as it potentially limits the usefulness of AI systems for the analysis of legal cases, e.g. for providing predictive advice as to sentencing decisions [27,28].

In this paper, we explore statistical techniques for understanding the forms of gendered differences or biases in legal judgments, with the aim of clarifying the gendered nature of these biases, to help legal scholars understand the prevalence and extent of unjust biases over a large body of legal cases. We focus on biases related to gender in legal judgments, where a *judgment* refers to a whole document, often over 100 pages, summarizing a case, setting out the background context, summaries of witness statements, citations of prior legislation, the reasoning and conclusions, including intermediate conclusions, made by the judge(s), and the decisions (or orders) made by the judge(s). Accordingly, the text of a judgment is minimally structured (into paragraphs), and sometimes uses formal linguistic constructions. However, it differs markedly from everyday speech.

Legal judgments can vary widely in length and complexity and while there are common features across judgments (and legal proceedings), the judgment texts do not follow a fixed format, and even though they will contain common elements or features, the identification and classification of the these features will be frequently contested [5].

Written judgments will also include many different ‘speakers’ as a judgment will contain not only the decision made by the judge and their reasons, but also may extract words spoken by witnesses, text from expert reports, sections of legislation, extracts from previous judgments (precedents) as well as reproducing submissions made by the parties, or made on their behalf by their legal representatives. Further, the language (or jargon) used is often specialized, utilizes formal or technical definitions that may or may not correspond to ‘ordinary’ usage, and decisions or rulings will often be implicit, highly nuanced and context-dependent.

All of these features make automated analysis and classification challenging. Due to this complexity, most analysis of language use in judgments, and in legal discourse more broadly, has used traditional content or discourse analysis [29], e.g. the analysis of judicial appointment speeches in McLoughlin [15]. Some recent work has considered ways to “scale up” traditional content analysis, for example using corpus techniques such as colocation and frequency tools. However, such work has tended to focus on small sets of cases, such as sentencing remarks in homicide cases where the gender of the parties (in this study the defendant) is clear [30].

There has also been a trend towards incorporating computer assisted corpus linguistics into legal interpretation tasks [31–33]. But the aim or focus of this computer-assisted statutory interpretation sits within a legal hermeneutics framework, and this form of “empirical textualism” is itself prone to replicating hidden biases [34]. Some groundbreaking work on analysing the outcomes in a large set of cases in refugee decision making was able to extract some limited information from decisions, but restricted the pool of cases to single issue claims where the outcome was clearly signalled in the court orders and where the range of possible outcomes was more akin to a ‘closed set’ [35]. That study did not attempt any analysis of the language used within the decisions and, for example, was unable to reliably determine information such as the country of origin of the applicant from the decision text. Finally, in some novel research reported recently, UK based herEthical.AI is working to develop a tool that will be able to identify victim-blaming language in police reports and legal judgments involving allegations of domestic and family violence [36,37]. The findings so far reported from this paper are consistent both with past research on language and attitudes in cases involving allegations of gendered violence, and with the discourse analysis undertaken as part of this project.

We restrict our study to cases from Australian family law, as there are often multiple parties to any dispute, clearly identifiable by gender, and where biases (unconscious or otherwise) are likely to be evident in the text of the judgment. We are particularly interested in how male and female parties or witnesses are treated differently when spoken to and about by judges, how their claims are assessed for validity or reliability (relating to their credibility), and how legal language reinforces or breaks down stereotypes, and contributes to the outcomes of the cases.

Our work builds upon numerous studies by feminist scholars highlighting gender biases in legal thinking, including in family law. Using the probabilistic topic modelling approach [38] to identify salient topics from the corpus, and then the structural topic model [39] to identify drivers of topics, our contribution is to apply these statistical methods at scale to determine the form and extent of biases over a large set of cases ( 2530 cases) over a long period of time (around 20 years), and to semi-automatically detect biases based on gender from the judgments themselves; this helps answer the question of the extent that the biases are present in the language of the judgments, or are to be found in the interpretation of the judgments by legal scholars. In fact, as we will show, gender-based language biases are clearly evident in the legal judgments and moreover, our analysis, being at the fine-grained word level, sheds light on the nature of such biases, both those already identified in the literature, and new forms of bias.

As part of the analysis, because we also have access to the gender of the judges (and typically the cases we study have only one judge), we can also determine if there are gender differences between male and female judges that are expressed in the text of the legal judgments – and indeed there are such differences. In addition, we can also detect changes over time in the expressed biases.

The legal judgments and the language used are too diverse for gender differences to appear at a document level. Therefore we focus in this paper on one prominent aspect of family law cases, *capacity*, as the capacity of the father/mother to provide care in the interests of the child is a major decision criterion for assessing the case and determining orders for access to the child and contributions to the child’s upbringing (many of the cases are about how to apportion care of the children). For the analysis, we first automatically extract those sentences (and sentence fragments) that are about capacity, along with the gender of the “target” of the language, typically the father or mother. This provides a subset of the legal text for more fine-grained analysis. We use structural topic models [39] to group this language into “topics”. We then perform statistical analysis to identify statistically significant differences between male–female language, which demonstrates potential biases in the text of the legal judgments. The topics can then be used as a concrete basis to ground discussion of the ramifications of the potential biases found.

## The Family Law context in Australia and bias in Family Law decision making

Family law (or more specifically matters pertaining to marriage, divorce and children of a marriage) is an area of Federal or Commonwealth responsibility under the Australian Constitution (see s 51 (xxi) and s 51 (xxii)). Under a combination of Commonwealth laws and the cross-vesting of state powers, the resolution of disputes about parenting of children after separation (including de facto separations) is governed primarily by the *Family Law Act 1975* (Cth) [40, Para 2.42–2.64]. Because of an emphasis on mediation and alternative dispute resolution (ADR) only a very small percentage of disputes will reach the stage of a formal court hearing and judgment (see below). Thus, the judgments we analysed in this research are those where the parties have been unable to resolve arrangements for parenting via informal agreement or court supported mediation or ADR.

Previous research has indicated that these cases are more likely to involve allegations of violence or abuse and/or where one parent is seeking to make a significant change to parenting arrangements, including moving to a new geographically distant location.

It is also worth noting that there have been four major tranches of amendments made to the Part of the *Family Law Act* governing parenting arrangements since 1975. These amendments reflect policy shifts driven in part by changing approaches to, and understandings of, child rights, the gendered nature of violence, as well as a greater expectation of and emphasis on shared parenting post separation. Some changes, particularly those implemented in the 1990s under the conservative Coalition government, were driven by successful advocacy from ‘Father’s Rights’ organizations [41–43].

As noted in more detail below, these tranches of reform ushered in new terminology to describe parenting orders as well as policy shifts aimed to support equal shared parental responsibility for decision making in relation to children, to encourage shared care arrangements and to prioritize maintaining children’s contact with both parents, even in situations where there was a history of family violence, or allegations of abuse [44]. For example, changes in 2006 reinforced expectations about shared care and equal parental rights and responsibilities, driven in part by a perception that fathers were missing out, but also introduced a primary consideration relating to protecting children from harm and abuse, neglect or family violence. Reforms in 2012 moderated some of the 2006 changes, including repealing a provision that had made it more difficult for parents, primarily mothers, to resist or restrict contact with a potentially violent or abusive father. The 2012 reforms also strengthened expectations that courts would take into account the impacts of family violence on children [41,45]. The most recent reforms, which have come into force in 2024, continue this trend, further shifting the emphasis away from presumptions about equal parenting and have revised the factors to be taken into account, including family violence, when determining the parenting orders that will be in the best interests of the children involved [46].

If we turn to some of the features or patterns in family law decision making in the courts in Australia, the first point to note is that only a very small proportion of cases actually come to court for formal resolution after a full hearing [41]. That is to say, the vast majority of parents come to an agreement about parenting arrangements. Statistics from the Australian Institute of Family Studies [47] indicate that the court is the main pathway for only 3% of separated parents, and only 16% come to arrangements after using a family dispute resolution service or lawyers. This study also shows that the majority of children reside primarily with the mother after separation but have regular contact with the father. Mothers have sole responsibility in 27% of cases, with fathers having sole responsibility in only 2% of cases. These patterns could be seen as a bias towards mothers, and against fathers, in the family law system, and as noted, some of the reforms to the family law system have responded to these stated concerns. However, the statistics need to be contextualized against the unequal division of childcare and household labour within families prior to separation [48]. We pick up on the significance of the background patterns of the division of household labour, and the impact of gendered expectations and norms that may flow from this in the decisions in our dataset, in our Discussion section below.

## Material and method

### Data collection and processing

#### Legal documents:

We restricted our analyses to judgments addressing parenting or custody disputes from the Family courts in Australia from the period 2001 to 2021. This is simply the period over which most of the judgments were available to us in digital form. This encompassed decisions across all levels of specialist Family Courts, including single judge decisions (single judge decisions are usually ‘trial level’ decisions from the Federal Circuit and Family Court or the Family Court) as well as appellate decisions from the Full Court of the Family Court (appellate courts are constituted by three to five judges).

As a result of the reform process referred to above, the terms used in legislation to describe types of parenting orders have changed over the years. Search terms used in this study for selecting the cases were determined by reference to the changing terminology. For example, the term ‘custody’ was replaced by ‘residence’ and ‘access’ by ‘contact’ in 1995, alongside other reforms to terminology that heralded a shift in expectations towards a model of shared parenting responsibility. Additional search terms were added (such as ‘independent child representative’) to ensure we were capturing as many cases concerning disputes relating to children/parenting as possible. We also included some additional terms to capture cases where there were allegations of family or domestic violence alongside parenting disputes. In all, we searched across the following catchwords and summaries, identifying all cases that had at least one of these phrases: ‘custody’ or ‘residence’ or ‘access’ or ‘contact’ or ‘parenting order⋆’ or ‘parenting plan⋆’ or ‘independent child⋆ lawyer’ or ‘independent child⋆ representative’ or ‘child support’ or ‘child maintenance order’ or ‘post-separation parenting program’ or ‘family violence order’, where ⋆ indicates a truncation wildcard (so that e.g. child⋆ matches child, children, child’s etc). We then restricted the dataset to those full text cases applying the *Family Law Act 1975 (Cth)*. In total, 3157 family court cases were obtained.

In single judge decisions, the name of the authoring judge is recorded, and in some Full Court cases the named judges delivered their reasons in single authored judgments. To ascertain the judge’s gender, we cross-referenced information from the Federal Circuit and Family Court of Australia (2024) via their official website (https://www.fcfcoa.gov.au) The gender of judge was determined from a combination of the judge’s name and photo, and personal knowledge. A small number of judgments were jointly made between male and female judges; in these cases, we do not analyse the judge gender aspect of these documents. This yielded 87 male judges and 53 female judges. We did not include any identifying features (e.g. names or titles) of judges in the analysis, focusing solely on their gender for analytical purposes.

The judgment documents are long, with the 3157 cases amounting to 351,140 paragraphs, consisting of catchwords, opinions, judgments and orders. The bulk of an opinion usually includes plaintiff’s and defendant’s arguments, their supporting cases, review, statutes, facts, etc. As we are interested in a judge’s own opinions and comments, we excluded the parts of documents (or excerpts) where prior evidence or reports from psychologists, previous court decisions, etc., were reproduced. A combination of custom functions and spaCy (https://spacy.io/), a Natural Language Processing library, was used to identify speakers and their roles from the legal text. First-person pronouns (such as ‘I’, ‘me’, ‘my’, etc.), and subject-verb relationships were examined to identify the main speaker and their statements for each paragraph.

#### Capacity subset:

Based on the structured categorization of data and word frequency analysis, we identified that the term ‘capacity’ was particularly important (appearing with high frequency in most cases) for its relevance in several key areas. We note that while the term ‘capacity’ itself is neutral, its meaning is multifaceted. This includes each parent’s capacity to make decisions (informed decisions regarding their own welfare or the welfare of their children), capacity to care for a child or children, capacity to work or financial capacity. It may also include (though less likely be the case in this data set) discussion of legal capacity to participate in legal proceedings, or consent to various legal agreements in question. However, we also know that the assessment of the past and future, anticipated, conduct of the parties is likely to be affected by gendered assumptions and expectations about parental roles and responsibilities (see discussion above). The prominence of the term ‘capacity’ is consistent with the overarching nature of cases where the core issue is a dispute over which parent is able to provide care that is in the ‘best interests of the child’. The parent’s ability or capacity to provide for the needs of the child is one (of many) of the considerations included to be considered (see [41] and ss 60CA and 60CC of the current iteration of the Family Law Act 1975), but is not a keyword that would necessarily have been able to be identified as a key term based on traditional discourse analysis methods. Consequently, we focused this study on how capacity is assessed and whether gender interacts with the language used within this setting. Excluding cases in which capacity was not a factor, specifically, where the case did not contain any of the keywords ‘capacity’, ‘ability’, ‘capable’, ‘able’, ‘capability’, ‘competency’, ‘skill’, or ‘quality’, and retaining only paragraphs that included at least one sentence for which the judge was determined to be the speaker (by virtue of it containing a first person pronoun), brought the total number of family court cases to 2700, encompassing 23,786 paragraphs containing 95,780 sentences.

#### Gender categorization:

Of the paragraphs determined to be a judge’s opinion, each paragraph was broken down into sentences, with compound sentences further split into sentential clauses, to facilitate a focused analysis of gender in 102,847 *sentential units*. In this process, each sentential unit within a paragraph was categorized with one of four distinct labels based on the types of nouns and pronouns that denote the targets of the language. Sentential units exclusively featuring female-specific nouns or pronouns, such as ‘she’, ‘her’, ‘mother’, were labelled ‘female’, while those with only male-specific nouns or pronouns, such as ‘he’, ‘his’, ‘father’, were labelled ‘male’. We use ‘male’ and ‘female’ as adjectives that designate a person’s gender identity as indicated by the language used in the judgments. Coreference resolution in spaCy was used to ensure that the pronouns refer to the parents rather than to lawyers, witnesses, children, etc. Sentential units containing nouns or pronouns from both genders were labelled ‘both’, while those without any gender-specific nouns or pronouns were labelled ‘unknown’. Sentential units without a clear assignment of gender were excluded from the analysis. After this stage of preprocessing, there were 21,137 sentential units labelled ‘female’, 23,315 labelled ‘male’, 29,970 labelled ‘both’ and 28,425 labelled ‘unknown’.

#### Extraction of judge statements and validation:

The last preprocessing step was to exclude paragraphs where the judge is not expressing an opinion or explaining their reasoning, specifically paragraphs headed by the terms ‘section’, ‘report’, ‘revised’ or ‘application’ (or their plurals), etc. This step was done last to facilitate reuse of the pipeline in other applications. Finally, a random sample of the extracted and labelled sentential units was manually checked to validate the correctness of data preprocessing.

#### Metadata integration:

Each judgment document contains the judge’s opinions and the gender of the targets for each sentential unit. Additional meta-information provides the gender of the judge and year of judgment. To limit any potential effects produced by longer vs shorter judgments, all of the ‘male’ sentential units from each case were combined into a single larger “document” for statistical analysis, and similarly for all the ‘female’ sentential units. In this way, each case generated either one or two documents for further analysis using topic modelling. The final dataset contains a total of 4330 such documents derived from 2530 cases, each labelled with the gender of the judge and the target, resulting in 2198 documents labelled ‘male’ and 2132 as ‘female’ for targets, while 1408 were associated with female judges and 2817 with male judges, with 105 documents where the speaking judge’s gender was unclear.

### Topic modelling

The latent Dirichlet allocation (LDA) model [38] is one of the most popular probabilistic methods for automatic separation of topics in a corpus. This is an unsupervised mixed membership model that allows each document to take on multiple topics, unlike other clustering techniques. LDA is a three level hierarchical Bayesian model, where each document is modelled as a finite mixture over a latent set of topics and each topic is modelled as a mixture of topic words (and other selected bigrams and trigrams). The topic probabilities assigned are a numerical representation of the original qualitative data indicating the likelihood of the corresponding topic. LDA preserves the necessary statistical relationships by identifying short descriptions of the members of a collection that enable efficient processing of large collections, that can then be used for basic tasks involving detection, classification and summarization of text. When covariate information is available, such as the gender of the judge and gender of the targets, and interest lies in how these covariates are related to each topic or word prevalence, the structural topic model (STM) [39,49], which extends LDA to incorporate covariates, is an appropriate model to use. The STM allows us to determine statistically significant factors influencing separate topics.

The STM incorporates the covariates to LDA at both the topic and word level, by modelling the topic proportions (or topical prevalence) parameters *θ* and word frequency (or topical content) parameters *β* as functions of covariates. Variational approximation and an expectation-maximization (EM) algorithm formed the basis for empirical Bayes parameter estimation. The stm package in R [50] was used to carry out model fitting. A detailed description of the STM is given in Supporting information (Methods I).

We consider only the topic level (topic prevalence) covariates, which were judge gender (‘male’ or ‘female’), target gender (‘male’ or ‘female’), judge and target gender interaction, and year of the judgment. While it is also possible to consider word-level covariates, we decided not to fit the model with these extra covariates, since interpretation at word level is much more difficult than at the topic level. Thus under the STM, each cleaned judgment document corresponds to a mixture of topics where the prevalence with which a topic is discussed and the content of the words used in a topic can alter based on the characteristics of the judgment document. The model allows us to make inference on the statistical significance of the covariates on the topic prevalence, hence allowing us to draw conclusions on the correlation of topic prevalence and covariates of interest (e.g. gender of judge). Additional details of the STM, including details of its implementation, is given in Supporting information (Methods II).

### Ethics statement

All of the judgments analysed in this study are public documents and are relied on and referenced in line with conventional legal citation and source attribution. In the case of family law judgments involving decisions relating to children, all parties have been de-identified by the court prior to publication (by way of pseudonyms). Judgment texts, which must include the name of the judge(s) making the decision, are sources of law, and as such are publically available, including on open databases (such as AustLII) and commercial databases (such as the LexisNexis^®^ database). No separate ethics approval is required to utilize or critically analyse judgments.

## Results

Table 1 shows the results from 12 topics together with their high probability words (HighProb) and their frequent and exclusive (FREX) words. While the HighProb words indicate most frequently co-occurring words, FREX indicates distinctive words which are more likely to occur *only* in the given topic, which are useful in distinguishing between the topic contents. Also shown in the table is the topic prevalence, given in percentages. Covariates (statistically) significantly (up to 0.1 significance level) associated with each topic are also shown; full details on the parameter estimates can be found in Table 2. The topic labels were manually assigned based on the highest probability and FREX words, along with the analysis of the context within the documents. The number of topics was determined via an exhaustive search across a sequence of values, see Supporting information (Methods II).

**Table 1:** Topics identified by STM, columns show topic label and their prevalence (in %), top 10 highest probability (HighProb) words and most frequent and exclusive words (FREX), and the statistically significant (P-value < 0.1) covariates.

Topic Label	HighProb	FREX	Association
**1. Evidence, Cross-examination (8.96%)**	evidence, accept, give, cross, report, examination, cross.examination, issue, court, witness	cross, cross.examination, examination, witness, give.evidence, answer, examine, expert, report, oral.evidence	target gender; judge gender; decision year
**2. Time, Capacity, Behaviour and Father’s Engagement (8.82%)**	father, child, time, spend, time.father, relationship, contact, would, spend.time, capacity	father, spend.time.father, time.father, child.father, father.say, accept.father, satisfied.father, father.would, father.evidence, father.capacity	target gender; decision year
**3. Co-parenting Arrangements (9.97%)**	child, school, care, parent, time, need, contact, day, relationship, arrangement	school, weekend, holiday, school.holiday, presently, night, care, day, old, care.child	target gender; judge gender; decision year
**4. Marital Finance and Property Assessment (5.41%)**	wife, property, child, party, work, make, income, home, contribution, financial	wife, contribution, adjustment, property, superannuation, matrimonial, favour, accept.wife, asset, matrimonial.home	target gender; judge gender; decision year
**5. Parental Responsibility and Best Interests of Child (7.10%)**	child, order, good, interest, good.interest, time, parent, make, spend, responsibility	parental.responsibility, good.interest, child.lawyer, independent.child, independent.child.lawyer, equal, child.good.interest, parental, interest, child.good	judge gender; decision year
**6. Decision Making and Care for Child (8.84%)**	mother, child, evidence, capacity, need, time, mother.evidence, provide, maternal, grandmother	accept.mother, mother.evidence, mother, mother.say, evidence.mother, mother.able, mother.would, time.mother, mother.child, mother.capacity	target gender
**7. Judicial Reasoning Process (7.16%)**	submission, reason, honour, make, counsel, evidence, finding, appeal, trial, case	honour, appeal, submission, judge, trial, ground, counsel, error, primary.judge, finding	judge gender; decision year
**8. Risk Assessment, Abuse and Family Violence (5.03%)**	child, risk, abuse, family, unacceptable, harm, unacceptable.risk, violence, make, allegation	unacceptable.risk, unacceptable, abuse, risk, harm, sexual, risk.child, risk.harm, family.violence, violence	target gender; decision year
**9. Communication, Expression, Behaviour with Child (8.86%)**	say, child, would, see, family, tell, time, one, go, want	go,tell, want, thing, say, get, ask, like, something, speak	judge gender
**10. Decision Making for Change in Circumstance (8.67%)**	would, able, likely, however, accept, live, return, view, australia, seem,	australia, travel, omit, country, move, return, sydney, remain, likely, new	judge gender; decision year
**11. Finances and Liability for Child Support (8.71%)**	husband, income, pay, support, financial, child.support, child, per, property, make	husband, child.support, income, pay, business, liability, payment, financial, tax, company	target gender; judge gender; decision year
**12. Compliance with Court Orders (12.46%)**	order, make, application, court, reasonable, seek, applicant, respondent, proceeding, order.make	contravention, applicant, excuse, reasonable.excuse, respondent, file, application, comply, reasonable, order.make	target gender; decision year

**Table 2:** Estimate of the coefficient for covariates of each topic, * indicates interaction terms. Only statistically significant (up to significance level of 0.1) are shown.

Topic	Covariate	Coefficient	P-value
**1. Evidence, Cross-examination**	female target	0.014	0.015
	female judge	-0.011	0.076
**2. Time, Capacity, Behaviour and Father’s Engagement**	female target	-0.157	0.000
	decision year	0.001	0.065
**3. Co-parenting Arrangements**	female target	-0.013	0.024
	female judge	0.0232	0.000
	decision year	-0.0051	0.000
**4. Marital Finance and Property Assessment**	female target	0.087	0.000
	female judge	0.018	0.023
	target (F) * judge (F)	0.025	0.048
	decision year	-0.001	0.015
**5. Parental Responsibility and Best Interests of Child**	female judge	-0.011	0.096
	decision year	0.002	0.000
**6. Decision Making and Care for Child**	female target	0.161	0.000
**7. Judicial Reasoning Process**	female judge	-0.021	0.001
	decision year	0.003	0.000
**8. Risk Assessment, Abuse and Family Violence**	female target	-0.011	0.047
	decision year	0.003	0.000
**9. Communication, Expression, Behaviour with Child**	female judge	-0.002	0.003
**10. Decision Making for Change in Circumstance**	female judge	-0.020	0.002
	decision year	-0.003	0.000
**11. Finances and Liability for Child Support**	female target	-0.091	0.000
	female judge	0.033	0.000
	target (F) * judge (F)	-0.029	0.027
	decision year	-0.002	0.002
**12. Compliance with Court Orders**	female target	0.011	0.099
	decision year	0.001	0.003

In order of topic prevalence, the topic “Compliance with Court Orders” (topic 12, 12.46%) is by far the most likely discussed topic by judges. This is followed by “Co-parenting Arrangements” (topic 3, 9.97%).

These are then followed by the following topics which all have similar prevalence rates of between 8.67%–8.96%. They are: “Evidence, Cross-examination” (topic 1), “Communication, Expression, Behaviour with Child”(topic 9), “Decision Making and Care for Child” (topic 6), “Time, Capacity, Behaviour and Father’s Engagement” (topic 2), “Finances and Liability for Child Support” (topic 11) and “Decision Making for Change in Circumstance” (topic 10).

“Judicial Reasoning Process” (topic 7) and “Parental Responsibility and Best Interests of Child” (topic 5) are slightly less prevalent at around 7%. Finally, topics , “Marital Finance and Property Assessment” (topic 4) and “Risk Assessment, Abuse and Family Violence” (topic 8) received around 5% relative coverage.

As shown in Table 2, topics 2 and 11 are “father” topics; the female target gender variable is strongly negatively associated (P-value around 0) with the topic prevalence. Under the topic “Time, Capacity, Behaviour and Father’s Engagement”, judges discuss fathers spending time, contact and engagement with a child. Topic keywords suggest a focus on the father’s interaction with the child, the nature of their relationship, and legal considerations such as visitation rights. There is also an emphasis on the unique aspects of the father’s engagement and satisfaction with the father-child relationship as perceived in legal settings. This topic has become significantly more prevalent over the years (P-value 0.065).

**Fig 1:**
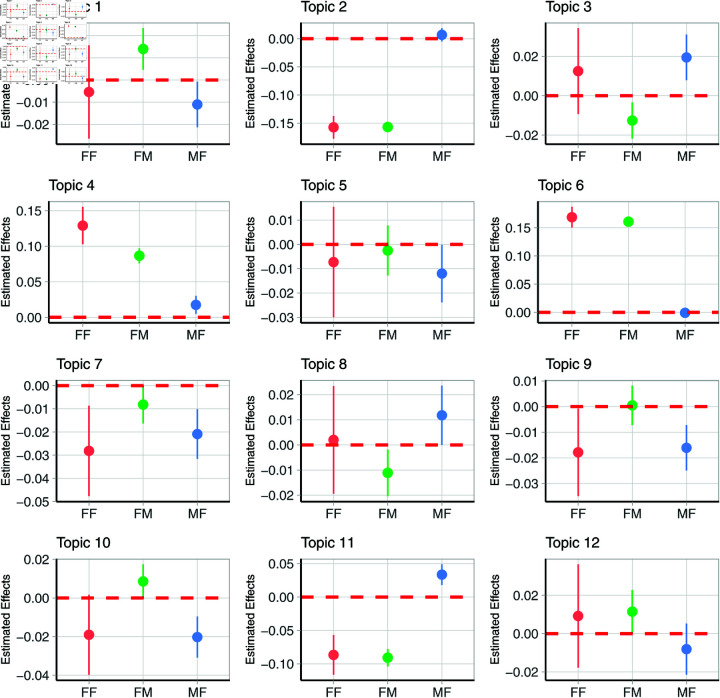
Comparison of the estimated effects across gender groups: female target and female judge (FF), female target and male judge (FM), and male target and female judge (MF), relative to baseline of male target and male judge (MM), which is shown as a dashed horizontal line. Vertical bars indicate the 90% confidence intervals, vertical bars not crossing the horizontal line are indicative of statistically significant effects from the baseline.

Judges also discuss “Finances and Liability for Child Support”, which concerns financial aspects, with financial obligations and support, particularly from the husband’s perspective. The topic also covers income, child support payments, and other financial responsibilities as part of divorce or separation proceedings. Interestingly, female judges are significantly more likely than male judges to discuss the mother’s financial position (see Fig 1), though such discussion is decreasing significantly over the years (P-value around 0.002), presumably as mothers become more financially self-sufficient.

In a similar way, topics 6 and 4 are “mother” topics. In “Decision Making and Care for Child”, judges discuss the mother’s abilities and responsibilities in child-rearing. The FREX terms such as ‘mother.evidence’ and ‘mother.able’ signifies the mother’s competence and possible evidence to support it. This topic prevalence has remained stable over time.

Another “mother” topic is “Marital Finance and Property Assessment”, which appears to cover financial matters in the context of marital separation or divorce, focusing on the division of assets, property, and income. FREX words like ‘matrimonial.home’ and ‘wife.evidence’ indicate specific legal arguments and evidence presented by or about the wife regarding financial contributions. Female judges are significantly more likely to discuss this topic, and particularly when this topic is discussed in the context of the “mother”. However, discussion of this topic is decreasing significantly over time. Fig 1 clearly shows the preference towards female targets for these two topics, where interestingly, similarly to the financial topic strongly associated with fathers (topic 11), female judges are again more likely to discuss the finance topic for female targets.

In addition to being more likely to discuss finances (topics 4 and 11), female judges are also more likely than male judges to discuss the topic of “Co-parenting Arrangements” (topic 3), and this is driven by female targets. The high-probability words indicate a concentration on the logistics and aspects of shared parenting, specifically how parents manage childcare, school activities, and daily routines. The FREX words highlight specific issues related to managing holidays and daily child care or the structure of co-parenting. This topic addresses the complexities and the practical aspects of parenting post-separation or divorce, considering the best interests of the child. This topic occurs more frequently when discussing fathers, however the topic prevalence is decreasing over time. Finally, female judges are more likely to touch on “Risk Assessment, Abuse and Family Violence” when discussing female targets (topic 8), (see Fig 1. The topic explores the serious issues of the domestic environments and the associated risks. The FREX words, particularly ‘unacceptable.risk’ and ‘sexual.abuse’, highlight specific concerns about the safety and well-being of children within the households. Again, the topic is addressed more frequently when discussing fathers. The topic has become more prevalent over time.

When male judges are discussing parental issues, they are more likely to discuss “Parental Responsibility and Best Interests of Child” (topic 5) which is primarily driven by male judges discussing fathers, and on “Communication, Expression, Behaviour, with Child” (topic 9). Male judges are also more likely to discuss “Evidence and Cross-examination” (topic 1), and mothers are more likely to be associated with this topic, suggesting that perhaps mothers are more likely to be asked to produce evidence. This is further seen with “Compliance with Court Orders” (topic 12), again appearing more with mothers, suggesting that mothers are held to a higher standard.

“Judicial Reasoning Process” (topic 7) focuses on formal presentation during court arguments, with FREX words such as ‘appeal’, ‘judge’ and ‘primary.judge’ indicating a particular focus on the process and the roles of judges in reviewing cases. Male judges are more likely to focus on this topic, and it is also associated more with fathers. This topic is increasing in prevalence over time.

For the topic “Decision Making for Change in Circumstances” (topic 10), the terms such as ‘live’ and ‘australia’ suggests the labelling. FREX words such as ‘travel’, ‘move’ and ‘return’ further emphasize custody and living arrangements that may be affected by moving to different locations, including interstate or international moves. This topic is becoming less prevalent and is seen more frequently with male judges.

In summary, discussions for mothers are more frequently associated with topics 1, 4, 6 and 12. These cover marital finances; ability to provide care and an emphasis on evidence. Discussions for fathers were more frequently associated with topics 2, 3, 8 and 11. These topics cover father’s interaction with child, logistics of parenting arrangements, domestic violence environments and financial obligations and support.

Female judges are more likely to discuss topics 3, 4, and 11, male judges are more likely to discuss a wider range of topics 1, 5,7, 9 and 10. Female judges tend to pay more attention to finances in relation to mothers, regardless of the type of finance (marital finance in topic 4 or child support in topic 11). Male judges tend to emphasize evidence, particularly in relation to mothers (topics 1 and 12).

## Limitations

In our methodological framework for topic modelling, as implemented in the structural topic model (STM), we differentiated between the gender of the target (‘male’ or ‘female’) and the gender of judges (‘male’ or ‘female’). This distinction was particularly relevant to understand the interplay between gender-specific language in legal documents and the gender of judges. However, we had methodological difficulties when processing sentences that involved comparisons between genders. For example, in the sentence pair: ‘He is not as impressive a witness as is the mother. He is at times quite verbose and some of his answers seem somewhat self-serving and very long.’, we focused entirely on the second sentence for gender-specific analysis as it unambiguously refers to a male individual. In contrast, the first sentence, which draws a comparison between a male and a female target, was categorized as ‘both’ due to its inclusive reference to both genders. This categorization approach reflected a deliberate methodological choice and was necessary for this initial study to isolate gender-specific language patterns. Future refinements may include more sophisticated handling of comparative sentences to understand comprehensive gender portrayals and their impact on judicial processes. We also note that not all decisions made by courts in Australia are made available in open or commercial legal databases, particularly from the earlier time period in our study, so we do not claim that our dataset includes all decisions made during the period covered by our analysis. Finally, while this was not a factor we were able to explore in this study, it is also important to note that many of the gendered expectations expressed in law are culturally specific. In Australia, for example, expectations about suitable family forms and parenting roles (such as the emphasis on a nuclear or heterosexual family unit) will play out in different ways for Aboriginal and Torres Strait Islander families [51], or if one or both of the parties identifies as non-heterosexual [52].

## Discussion

This study adds a new dimension to the body of feminist legal scholarship addressing the question of whether increasing diversity in the legal profession, and in particular diversity on the bench, can or will make a difference to the development, implementation and practice of law [13,53,54]. As noted in the introduction, legal texts in the form of judgments are constitutive, not merely reflective, and Common law systems, including Australia’s, have a masculinist history. Women were explicitly excluded from the legal profession in Australia until the early twentieth century (the last State to permit women to enter the legal profession was Western Australia in 1923 [55]). Other social, racial or other minority groups, also faced a combination of formal and informal barriers to entry [56]. This also has had an impact on the diversity of the profession, but to date the data beyond gender is lacking [57]. Although there are no longer formal barriers to entry into the profession for women, this history of exclusion contributes to the ‘masculinist’ character of the legal profession and to diminishing diversity as one looks up the judicial and professional hierarchy. For example, while female students outnumber males in law schools, and the majority of solicitors nationally identify as female, women remain under-represented in the Family Law Division of the Federal Circuit Court and on most superior courts [58]. This is reflected in Table 3, which shows the gender distribution across the documents in our dataset. As expected, the distribution between male and female targets is approximately even as most cases involve discussion of both mother and father, however the number of female judges is only half of the number of male judges. This is significant because precedent is written by higher or appellate courts. Thus, feminist legal scholarship has long advocated for increasing the representation of women in the judiciary, in part because this is seen to be a strategy for increasing the responsiveness of courts at trial level to the diverse experience of litigants and also for influencing the formation of more inclusive and unbiased legal rules and practice [15,59,60]. Further, as noted above, while only a small proportion of cases may come to the court, those that are resolved via negotiation, mediation or other formal and informal dispute resolution processes will be ‘bargaining in the shadow of the law’ [61,62]. Thus the legal frameworks developed in the case law will directly impact cases decided outside of those frameworks. The results in this study, highlighting several forms of gendered discrepancies to be found in the text of legal judgments, specifically concerning the factors that are considered in the context of assessing the capacity of parents, indicate that at the very least that the lived experience, and the gender identity, of the judges in our study do have an impact on their approach to decision making.

**Table 3:** Gender composition of the documents.

	Male Judge	Female Judge	Unknown	Total
**Male Target**	1437	711	50	2198
**Female Target**	1380	697	55	2132
**Total**	2817	1408	105	4330

In terms of the gender biases found in the legal judgments, some expected differences relating to societal gender roles are clearly evident (taking the time period analysed into consideration). In particular, financial concerns for fathers (topic prevalence of 8.71%) are more important than for mothers (topic prevalence of 5.41%), reinforcing the stereotype, or else conforming to societal expectations, however this difference is decreasing over time, presumably as women become more financially self-sufficient. It is also noticeable that female judges pay more attention to finances than do male judges. In contrast, more attention is given to the mother’s care-giving role, by both male and female judges. A feature of normative, gendered, expectations around childcare can include presumptions that mothers are better placed to offer care to younger children. And these gendered ideologies, and the realities of gendered divisions of labour that flow from them, may persist even as ideologies of ‘new fatherhood’ shift to include expectations that fathers will be more actively involved in childcare [63]. Or, conversely, fathers may receive greater recognition for their participation in family decision making or care when compared to mothers, based on an unexpressed assumption that family and household management, alongside quotidian childcare, are the responsibility of the mother. For example, a court accepted, as reasonable, a father’s justification of his ‘limited’ engagement in routine tasks such as supervising homework because he wanted to prioritize quality time with his child ([2020] FamCA 29). And a father’s involvement post separation will often be favourably recognized even where that participation is mediated or facilitated by a new female partner (see e.g. [2012] FamCA 1064 where the court was satisfied that the father was able to provide for the needs of his children ‘with the assistance of his present wife’. In terms of the outcome, the father is credited for the actions of his new partner, having provided the children with a ‘substitute carer’ [43] who had ‘relieved the father of responsibilities that should be his’ and had paid for school transport and medical treatment for his children. Despite gradual shifts (including an increase during COVID in the time fathers spent with their children [64]), there remain significant disparities in the division of household labour and parenting tasks, with women and mothers far more likely to be responsible for the majority of the routine childcare, as well as the less visible executive planning for the family, often while multitasking, whereas fathers are more likely to be engaged in supervising during (more visible) social or sporting activities [51,65]. These wider social patterns, as well as the history of an individual family’s arrangements, will necessarily impact arrangements post separation [51]. But there may be gendered bias in legal decision making if there is differential weighting accorded to involvement in ‘routine’ and ‘social’ parenting tasks. Despite statement of legal precedent on the importance of avoiding the use of gender stereotyping in decision making as far back as 1996 [66], gender norms are notoriously ‘sticky’ [15] and continue to have complex impacts on family law decision making [67]. Because of the multifaceted nature of the orders that are made at the conclusion of a case, it is not possible to draw conclusions that the differential language and approach, as between male and female judges, leads to a difference at the level of outcome in the decision. However, when these differences are considered alongside analyses of the prevalence and impacts of gendered language and decision making conducted via more traditional discourse/case analysis, these language differences (and the biases they seem to invoke) are noteworthy.

The analysis also produced some unanticipated results. For example, the co-parenting arrangements of the father are more often considered by female than male judges. Arguably this bias encourages fathers to take a more active, rather than transactional, role in their children’s upbringing. The observed increased attention paid by female judges to the risks of harm to the child could support the feminist contention that greater diversity on the bench will contribute to legal decision making that is more responsive to vulnerable parties, and more likely to recognize the social context and consequence of legal decisions. In family law disputes relating to children, analysis has focused on differential treatment of allegations of violence made by men and women [68,69], in addition to the differences in the ways in which the involvement and contributions of mothers and fathers are valued and rewarded [51], discussed above.

Finally, there are other marked differences between male and female judges. Male judges tend to talk more than female judges about court procedures and processes, especially in relation to female targets. We are unable to suggest any reason for this.

## Conclusion: Do female judges make a difference?

We conclude that there are significant gender differences in judges’ language, and differences in the language directed towards males and females, in some instances independent of the judge’s gender and in other instances dependent on the judge’s gender. The fact that we can demonstrate these results shows the efficacy of structural topic models for undertaking this type of analysis. However, what is not discernible with this analysis is whether there is any effect of these linguistic biases on the outcomes of the cases. This will be the focus of future work. The findings of this study are based on a single dataset in the Australian family court setting. Additional analyses, using different datasets and different methodology will be needed to further consolidate these findings. However, returning to the significance of legal language, where differences exist, it is important to note that these can operate both discursively and substantively. That is to say, because legal language has a performative dimension [5] and written judgments create and implement legal relations and obligations between parties, biases in the language used in legal texts, or by legal actors, can have real world consequences for equality and fairness, even when the legal rules or presumptions are putatively expressed in gender neutral terms [70].

## Supporting information

S1 File Supplementary material. (PDF)

## References

[1] Naffine N. Law and the sexes: explorations in feminist jurisprudence. Sydney: Allen & Unwin; 1990.

[2] Graycar R, Morgan J. The hidden gender of law. 2nd ed. Sydney: The Federation Press; 2002.

[3] Mossman MJ. The first women lawyers: a comparative study of gender, law and the legal professions. Oxford: Hart Publishing; 2006.

[4] High Court of Australia. PGA v The Queen [2012] HCA 21 (30 May 2012). Commonwealth of Australia; 2012.

[5] Davies M. Asking the law question. 5th ed. Australia: Thomson Reuters; 2023.

[6] Caldas-Coulthard CR, Moon R. ‘Curvy, hunky, kinky’: using corpora as tools for critical analysis. Discourse & Society. 2010;21(2):99–133.

[7] Harris AP. Race and essentialism in feminist legal theory. Stanford Law Review. 2018;42:581–616.

[8] Skewes L, Fine C, Haslam N. Beyond mars and venus: the role of gender essentialism in support for gender inequality and backlash. PLoS One. 2018;13(7):e0200921. doi: 10.1371/journal.pone.0200921 30040839 PMC6057632

[9] Cunliffe E. Murder, medicine and motherhood. Oxford: Hart Publishing; 2011.

[10] Thompson LE, Pozzulo J. How length of and reason for delayed reporting influence mock-jurors’ judgments in a sexual assault trial. Journal of Police and Criminal Psychology. 2024.

[11] Gleason SA, Smart E. You think; therefore I am: gender schemas and context in oral arguments at the Supreme Court 1979 –2016. Political Research Quarterly. 2023;76(1):143–57.

[12] Hunter R, McGlynn C, Rackley E. Feminist judgments: from theory to practice. Oxford: Hart Publishing; 2010.

[13] Douglas H, Bartlett F, Luker T, Hunter R. Australian feminist judgments: righting and rewriting law. Oxford: Hart Publishing; 2014.

[14] Berns S. To speak as a judge: difference, voice and power. Aldershot: Ashgate; 1999.

[15] McLoughlin K. Law, women judges and the gender order: lessons from the high court of Australia. Abingdon: Routledge; 2022.

[16] Mack K. Continuing barriers to women’s credibility: a feminist perspective on the proof process. Crim Law Forum. 1993;4(2):327–53. doi: 10.1007/bf01096078

[17] Daly E. Rape, gender and class: intersections in courtroom narratives. Cham: Springer; 2022.

[18] Graycar R. Hoovering as a hobby and other stories: gendered assessments of personal injury damages. University of British Columbia Law Review. 1997;31:17–35.

[19] Graycar R. Damaging stereotypes: the return of ‘hoovering as a hobby’. In: Richardson J, Rackley E, editors. Feminist perspectives on tort law. Abingdon: Routledge; 2012. p. 205–26.

[20] New York State Judicial Commitee on Women in the Courts. Gender survey 2020 . State of New York Unified Court System; 2020.

[21] Jacobi T, Schweers D. Justice, interrupted: the effect of gender, ideology, and seniority at Supreme Court oral arguments. Virginia Law Review. 2017;103(7):1379–496.

[22] Loughland A. Female judges, interrupted: a study of interruption behaviour during oral argument in the High Court of Australia. Melbourne University Law Review. 2019;43(2):822–51.

[23] Jacobi T, Robinson Z, Leslie P. Querying the gender dynamics of interruptions at Australian oral argument. UNSW Law Journal Forum. 2020.

[24] Reinharz S, Davidman L. Feminist methods in social research. Oxford: Oxford University Press; 1992.

[25] Sarmas L. Story telling and the law: a case study of Louth v Diprose. Melbourne University Law Review. 1993;19:701–28.

[26] Rathus Z. Of ‘hoods’ and ‘ships’ and citizens: the contradictions confronting mothers in the new post-separation family. Griffith Law Review. 2010;19(3):438–71.

[27] Matthews S, Hudzina J, Sepehr D. Gender and racial stereotype detection in legal opinion word embeddings. AAAI. 2022;36(11):12026–33. doi: 10.1609/aaai.v36i11.21461

[28] Babaeianjelodar M, Lorenz S, Gordon J, Matthews J, Freitag E. Quantifying gender bias in different corpora. In: Companion Proceedings of the Web Conference 2020 ; 2020. p. 752–9.

[29] Prior L. Content analysis. In: Leavy P, editor. The oxford handbook of qualitative research. 2nd ed. Oxford: Oxford University Press; 2014. p. 541–68.

[30] Potts A, Formato F. Women victims of men who murder: XML mark-up for nomination, collocation, and frequency analysis of language of the law. In: Angouri J, Baxter J, editors. The Routledge Handbook of Language, Gender, and Sexuality. Abingdon: Routledge; 2021. p. 602–18.

[31] Solan LM, Gales T. Corpus linguistics as a tool in legal interpretation. BYU Law Review. 2017;2017(6):1311–57.

[32] Mouritsen SC. Corpus linguistics in legal interpretation—an evolving interpretative framework. International Journal of Language & Law. 2017;6:67–89.

[33] Vogel F, Hamann H, Gauer I. Computer-assisted legal linguistics: corpus analysis as a new tool for legal studies. Law & Social Inquiry. 2018;43(4):1340–63.

[34] Jennejohn M, Nelson S, Núñez DC. Hidden bias in empirical textualism. Georgetown Law Journal. 2020;109(4):767–811.

[35] Ghezelbash D, Dorostkar K, Walsh S. A data driven approach to evaluating and improving judicial decision-making: statistical analysis of the judicial review of refugee cases in Australia. UNSW Law Journal. 2022;45(4). doi: 10.53637/tcnq8226

[36] Hall R. Family court judges use victim-blaming language in domestic abuse cases, finds AI project. The Guardian. 2024. https://www.theguardian.com/law/2024/oct/08/family-court-judges-victim-blaming-language-domestic-abuse-cases-ai-project

[37] herEthical.AI. [cited 2024 Dec 20]. https://www.herethical.ai/

[38] Blei DM, Ng AY, Jordan MI. Latent dirichlet allocation. Journal of Machine Learning Research. 2003;3:993–1022.

[39] Roberts ME, Tingley D, Stewart BM, Airoldi EM. The structural topic model and applied social science; Presented at the NIPS 2013 Workshop on Topic Models: Computation, Application, and Evaluation. 2013.

[40] Australian Law Reform Commission. Family Violence: A National Legal Response. Commonwealth of Australia; 2010.

[41] Fehlberg B, Kaspiew R, Millbank J, Kelly F, Behrens J. Australian family law: the contemporary context. 2nd ed. Oxford: Oxford University Press; 2015.

[42] Kaye M, Tolmie J. Discoursing dads: the rhetorical devices of fathers’ rights groups. Melbourne University Law Review. 1998;22(1):162–94.

[43] Kaye M, Tolmie J. Fathers’ rights groups in Australia and their engagement with issues in family law. Australian Journal of Family Law. 1998;12(1):19–67.

[44] Jeffries S, Field R, Menih H, Rathus Z. Good evidence, safe outcomes in parenting matters involving domestic violence? Understanding family report writing practice from the perspective of professionals working in the family law system. UNSW Law Journal. 2016;39:1355–88.

[45] Rathus Z. The repeal of Australia’s problematic family law presumption (and other amendments): cautiously welcomed – but what has been lost? Journal of Social Welfare and Family Law. 2024;1–21. doi: 10.1080/09649069.2024.2414622

[46] Attorney-General’s Department. Family Law Amendment Act 2023 : Factsheet for Family Law Professionals. Commonwealth of Australia. 2024. https://www.ag.gov.au/families-and-marriage/publications/family-law-amendment-act-2023-factsheet-family-law-professionals

[47] Australian Institute of Family Studies. [cited 2024 Dec 20]. https://aifs.gov.au/

[48] Baxter J. Towards COVID normal: sharing of housework in couple families. Australian Institute of Family Studies; 2021.

[49] Roberts ME, Stewart BM, Airoldi EM. A model of text for experimentation in the social sciences. Journal of the American Statistical Association. 2016;111(515):988–1003.

[50] Roberts ME, Stewart BM, Tingley D. STM: an R package for structural topic models. J Stat Soft. 2019;91(2). doi: 10.18637/jss.v091.i02

[51] Parashar A, Dominella F. The Family in Law. Cambridge: Cambridge University Press; 2017.

[52] Millbank J. The limits of functional family: Lesbian mother litigation in the era of the eternal biological family. International Journal of Law, Policy and the Family. 2008;22(2):149–77.

[53] Wilson B. Will women judges really make a difference?. Osgoode Hall Law Journal. 1990;28(3):507–22.

[54] Rebouché R. Feminist judgments: family law opinions rewritten. Cambridge: Cambridge University Press; 2020.

[55] Kirk LJ. Portia’s place: Australia’s first women lawyers. Australian Journal of Legal History. 1995;1:75–91.

[56] Dehm S. Legal exclusions: Émigré lawyers, admissions to legal practice and the cultural transformation of the Australian legal profession. Federal Law Review. 2021;49(3):327–51.

[57] Opeskin B, Roach Anleu S. Judicial diversity in Australia: a roadmap for data collection. Australian Institute of Judicial Administration; 2023. https://aija.org.au/publications/judicial-diversity-in-australia-a-roadmap-for-data-collection/

[58] Australian Institute of Judicial Administration. AIJA Judicial Gender Statistics 2024 . 2024.

[59] Hunter R. More than just a different face? Judicial diversity and decision-making. Current Legal Problems. 2015;68(1):119–41.

[60] Watson N, Douglas H. Indigenous legal judgments: bringing indigenous voices into judicial decision making. Abingdon: Routledge; 2021.

[61] Mnookin RH, Kornhauser L. Bargaining in the shadow of the law: the case of divorce. Yale Law Journal. 1978;88:950–97.

[62] Crowe J, Field R, Toohey L, Partridge H, McAllister L. Bargaining in the shadow of the folk law: expanding the concept of the shadow of the law in family dispute resolution. Sydney Law Review. 2018;40(3):319–38.

[63] Busby N, Weldon-Johns M. Fathers as carers in UK law and policy: dominant ideologies and lived experience. Journal of Social Welfare and Family Law. 2019;41(3):280–301.

[64] Baxter J, Budinski M, Carroll M, Hand K. Life during COVID-19: dads spend more quality time with kids. Australian Institute of Family Studies; 2020.

[65] Harland A, Cooper D, Turnbull C, Rundle L. Family law principles. 3rd ed. Australia: LawBook Co.; 2015.

[66] Vickers L. “There’s No Gender Bias Here!”: Gender equality and family court custody decisions - The legacy of McMillan v Jackson. Sister in Law. 1996;1:33–51.

[67] Alexander R. Gender issues in family law: the impact of gender on decision making in Australia’s family law system. Chatswood: LexisNexis; 2023.

[68] Mindthoff A, Goldfarb D, Behre KA. How social science can help us understand why family courts may discount women’s testimony in intimate partner violence cases. Family Law Quarterly. 2019;53(3):243–64.

[69] Rathus Z, Jeffries S, Menih H, Field R. “It’s like standing on a beach, holding your children’s hands, and having a tsunami just coming towards you”: intimate partner violence and “expert” assessments in australian family law. Victims & Offenders. 2019;14(4):408–40. doi: 10.1080/15564886.2019.1580646

[70] Rathus Z. Social science or ‘legoscience’? Presumptions, politics, parenting and the new family law. QUT Law and Justice Journal. 2010;10(2):164–90.

