# Supplementary material for: Diversity on the bench: An analysis of gendered biases in the language of Australian Family Law Court judgments
Source: PLoS One. 2025 Sep 8;20(9):e0331841. doi: 10.1371/journal.pone.0331841 (PMC12416720; doi:10.1371/journal.pone.0331841)
Supplement: S1 File — (PDF) [file pone.0331841.s001.pdf]

SUPPORTING INFORMATION

Methods

I. Structural topic model (STM)

The latent Dirichlet allocation (LDA) model [1] is a generative model for documents, specifying a probabilistic process by which documents are assumed to be created. For each individual word in the document, a topic is randomly selected based on a predefined distribution and a word is then drawn from that corresponding topic. This processes results in the generative model for each document d via the following steps:

1. Draw topic proportions $\theta_d \mid \alpha \sim Dirichlet(\alpha)$,
2. For each word
   (a) Draw topic assignment $z_{d,N} \mid \theta_d \sim Mult(\theta_d)$,
   (b) Draw word $w_{d,N} \mid z_{d,N}, \beta_{z_{d,N}} \sim Mult(\beta_{z_{d,N}})$,

where θ_d is the K dimensional vector representing the topic proportions of a document, drawn from a Dirichlet prior distribution with hyper-parameters α. Words ($w_{d,N}$) in each document are generated by first assigning it to a topic, where the topic indicator ($z_{d,N}$) is drawn according to topic proportions θ_d, followed by drawing a word from that corresponding topic via a multinomial distribution with parameters $\beta_{z_{d,N}}$, where $\beta_1, \ldots, \beta_K$ gives the distribution over the terms in the vocabulary associated with each topic $1, \ldots, K$. LDA uses a "bag of words" approach, where data is represented as vectors of word counts. Under this representation, the order in which the words are used is completely disregarded. Topic formation is therefore based on word co-occurrence.

The structural topic model (STM) [2,3] extends LDA by incorporating covariates at both the topic and word level, by modelling topic prevalence parameters θ and topic content parameters β as functions of the covariates. For topic prevalence, the Dirichlet distribution controlling topic proportions θ_d is replaced with a logistic normal distribution whose mean function is parameterized as function of the covariates:

$$\theta_d \sim \text{LogisticNormal}(\Gamma X_d, \Sigma)$$

where Γ is the matrix of coefficients for the corresponding topic-covariates X_d. For topic content:

$$\beta_{z_{d,N}} \sim \frac{\exp(m_N + \kappa_{z_{d,N}} Y_d)}{\sum \exp(m_N + \kappa_{z_{d,N}} Y_d)}$$

where m models the corpus-wide background rate and κ is the matrix of coefficients and Y the content covariates.

For each document the model outputs the topic prevalence which represents the proportion of words attributed to each topic from covariate X and the topical content captures the words most likely generated from each topic based on the covariates Y. The interpretation of the coefficients of model covariates is similar to that of logistic regression models. A positive coefficient parameter which is statistically significantly larger/smaller than 0 for a given covariate, whose coefficient is denoted by β, would

suggest that the presence of the covariate is associated with an increase/decrease in the portion of the judge's opinion spent discussing a certain topic. Moreover, the coefficient β has a log odds ratio interpretation, where one unit of change in the covariate equates to changes in the log odds of the relevant topic proportion θ.

II. STM Model Fitting and Optimization

An in-depth preprocessing and vectorization process was conducted on the text data to facilitate effective topic modelling. A custom tokenizer using `spacy` [4] and NLTK [5] with standard English dictionary was used to remove stopwords, punctuation and special characters. Subsequently, CountVectorizer from `scikit-learn` [6] was employed to create vectorizers for unigrams, bigrams and trigrams. These vectorizers generate a document-term matrix, quantifying the occurrence of each term in the corpus. The average unigram frequency per document was 44.91. We used this number as a threshold for selecting terms to include in the document-term matrix, so that in total the LDA model uses 1904 terms that cover the most frequently occurring words of the documents, plus a variety of commonly occurring bigrams and trigrams. Figure 1 shows the distribution of the terms that appear in at least 10 documents.

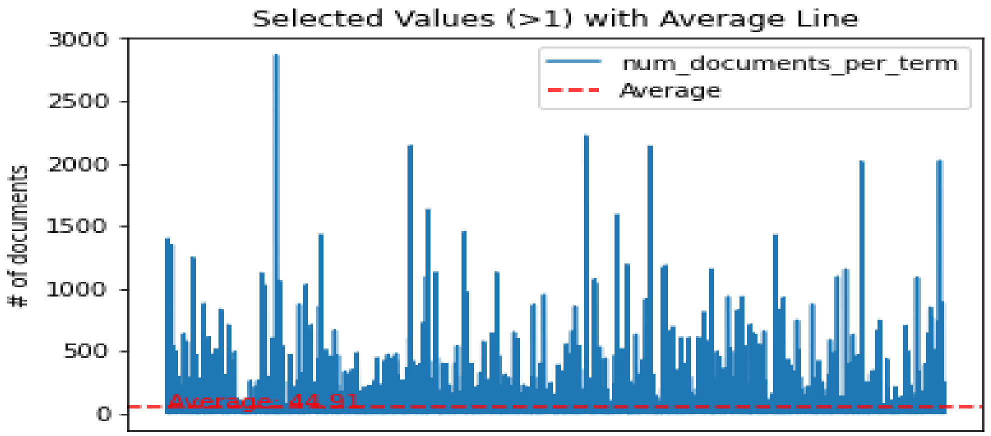

Fig 1. Distribution of the terms that appear in at least 10 documents within the corpus. The bars indicate individual terms, and their height shows the frequency of those terms across different documents. The red dashed line represents the average number of documents per term, which in this case is 44.91.

To fit a structural topic model, a crucial step is to determine the optimal number of topics that best represent the underlying structure of the data. The `manyTopics` function from the `stm` package [7] in R was employed to systematically test a range of topic numbers. Specifically, we systematically tested between 2 to 20 topics in increments of 2. The metrics *exclusivity* (distinctiveness of the topics) [8] and *semantic coherence* [9] were monitored.

Figure 2 shows the two metrics across 2 to 20 topics, with each metric rescaled to between 0 and 1 for visualisation purposes. The numbers of K between 10 to 15 represent a reasonable trade-off between exclusivity and semantic coherence of the topics. Note that choosing K too large results in too many topics and less coherence within topics, but choosing K too small results in some potentially important topics being missed. We decided to use $K = 12$ as a reasonable compromise to perform the analyses, the point at which exclusivity starts to stabilise (or even decline, as in Figure **??**) as the number of topics increases. After $K = 14$, Semantic Coherence decreases rapidly.

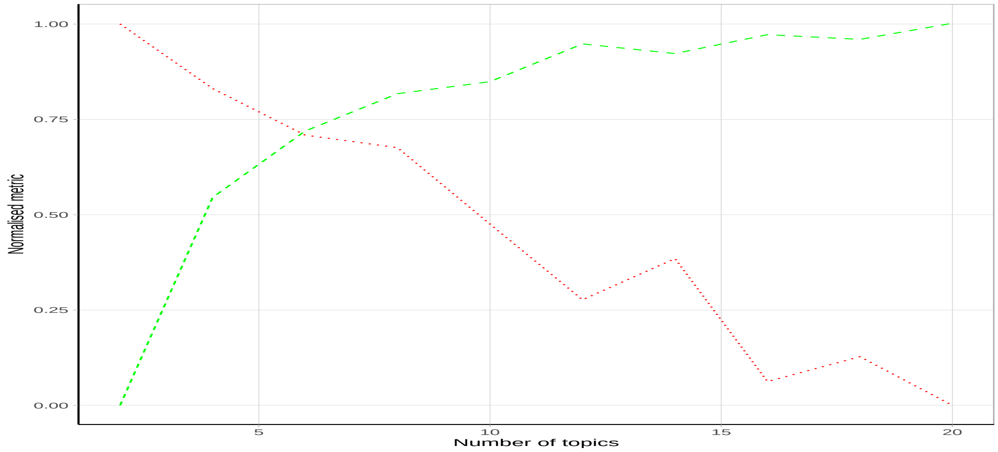

Fig 2. Normalised metrics as a function of the number of topics (K): Exclusivity (green dashed line) and Semantic Coherence (red dotted line).

References

1. Blei DM, Ng AY, Jordan MI. Latent Dirichlet Allocation. Journal of Machine Learning Research. 2003;3:993–1022.

2. Roberts ME, Tingley D, Stewart BM, Airoldi EM. The Structural Topic Model and Applied Social Science; 2013. Presented at the NIPS 2013 Workshop on Topic Models: Computation, Application, and Evaluation.

3. Roberts ME, Stewart BM, Airoldi EM. A Model of Text for Experimentation in the Social Sciences. Journal of the American Statistical Association. 2016;111(515):988–1003.

4. Honnibal M, Montani I, Van Landeghem S, Boyd A. spaCy: : Industrial-strength Natural Language Processing in Python; 2020. Available from: `DOI:10.5281/zenodo.1212303`.

5. Bird S, Klein E, Loper E. Natural language processing with Python: analyzing text with the natural language toolkit. " O'Reilly Media, Inc."; 2009.

6. Pedregosa F, Varoquaux G, Gramfort A, Michel V, Thirion B, Grisel O, et al. Scikit-learn: Machine learning in Python. Journal of machine learning research. 2011;12(Oct):2825–2830.

7. Roberts ME, Stewart BM, Tingley D. stm: An R Package for Structural Topic Models. Journal of Statistical Software. 2019;91(2). Available from: `http://www.jstatsoft.org/v91/i02/`.

8. Bischof JM, Airoldi EM. Summarizing Topical Content with Word Frequency and Exclusivity. In: Proceedings of the 29th International Conference on Machine Learning; 2012. p. 9–16.

9. Mimno D, Wallach HM, Talley E, Leenders M, McCallum A. Optimizing Semantic Coherence in Topic Models. In: Proceedings of the 2011 Conference on Empirical Methods in Natural Language Processing. Chicago: Association for Computational Linguistics; 2011. p. 262–272.
